# Exercise-induced inflammation alters the perception and visual exploration of emotional interactions

**DOI:** 10.1016/j.bbih.2024.100806

**Published:** 2024-06-11

**Authors:** Johannes Keck, Celine Honekamp, Kristina Gebhardt, Svenja Nolte, Marcel Linka, Benjamin de Haas, Jörn Munzert, Karsten Krüger, Britta Krüger

**Affiliations:** a Sensorimotor Control and Learning, Centre for Cognitive Science, Technical University of Darmstadt, Germany; b Neuromotor Behavior Lab, Institute of Sports Science, Justus-Liebig-University Giessen, Giessen, Germany; c Department of Exercise Physiology and Sports Therapy, Institute of Sports Science, Justus-Liebig-University Giessen, Giessen, Germany; d Department of Experimental Psychology, Justus-Liebig-University Giessen, Germany; e Center for Mind, Brain and Behavior (CMBB), Phillips University of Marburg and Justus-Liebig-University Giessen, Germany

**Keywords:** Emotion recognition, Eye-tracking, Social interactions, Point-light-displays, Inflammation

## Abstract

**Introduction:**

The study aimed to investigate whether an exercise-induced pro-inflammatory response alters the perception as well as visual exploration of emotional body language in social interactions.

**Methods:**

In a within-subject design, 19 male, healthy adults aged between 19 and 33 years performed a downhill run for 45 min at 70% of their VO_2max_ on a treadmill to induce maximal myokine blood elevations, leading to a pro-inflammatory status. Two control conditions were selected: a control run with no decline and a rest condition without physical exercise. Blood samples were taken before (T0), directly after (T1), 3 h after (T3), and 24 h after (T24) each exercise for analyzing the inflammatory response. 3 h after exercise, participants observed point-light displays (PLDs) of human interactions portraying four emotions (happiness, affection, sadness, and anger). Participants categorized the emotional content, assessed the emotional intensity of the stimuli, and indicated their confidence in their ratings. Eye movements during the entire paradigm and self-reported current mood were also recorded.

**Results:**

The downhill exercise condition resulted in significant elevations of measured cytokines (IL6, CRP, MCP-1) and markers for muscle damage (Myoglobin) compared to the control running condition, indicating a pro-inflammatory state after the downhill run. Emotion recognition rates decreased significantly after the downhill run, whereas no such effect was observed after control running. Participants' sensitivity to emotion-specific cues also declined. However, the downhill run had no effect on the perceived emotional intensity or the subjective confidence in the given ratings. Visual scanning behavior was affected after the downhill run, with participants fixating more on sad stimuli, in contrast to the control conditions, where participants exhibited more fixations while observing happy stimuli.

**Conclusion:**

Our study demonstrates that inflammation, induced through a downhill running model, impairs perception and emotional recognition abilities. Specifically, inflammation leads to decreased recognition rates of emotional content of social interactions, attributable to diminished discrimination capabilities across all emotional categories. Additionally, we observed alterations in visual exploration behavior. This confirms that inflammation significantly affects an individual's responsiveness to social and affective stimuli.

## 1 Introduction

Imagine a person walking towards you, expanding their chest, clenching and lifting their fists. Instantly, we may feel that the person we are looking at is filled with anger and that we should draw away from this potential threat. This example makes clear that humans are able to quickly retrieve a wealth of information through mere observation of a person's Emotional Body Language (EBL) (Atkinson et al., 2004; Bachmann et al., 2018; de Gelder et al., 2015; Keck et al., 2022; Poyo Solanas et al., 2020).

Despite the general ability to recognize emotions of conspecifics through EBL (de Gelder, 2006), it is assumed that human perception of the world and the people around is shaped by individual states and experiences (for a review, see Gallese, 2007) such as an individual's mood, gender or mental illness (e.g., Alaerts et al., 2011; Van Den Stock et al., 2007). In addition, bodily conditions like systemic inflammation can also influence perceptual functioning. Literature states that pro-inflammatory cytokines such as interleukin-6 (IL-6) and tumor-necrosis-factor-α (TNF-α) act as key mediators in the crosstalk between inflammatory pathways and neurocircuits in the brain influencing behavioral responses (Smith, 2000; Turnbull and Rivier, 1999). These cytokines activate the hypothalamus via different pathways (Dantzer et al., 2000, 2008; Dantzer and Kelley, 2007; Maier and Watkins, 1998) and, thereby influence the metabolism of serotonin and dopamine which play an important role in a variety of mood-, depression- and sickness related behaviours (Elenkov, 2008; Engler et al., 2016; Konsman et al., 2002; Miller et al., 2009; Schedlowski et al., 2014).

One of the most used research tools for investigating the relationship between mood, emotion perception and inflammatory states, is the experimental use of endotoxin, vaccinations or the direct injection of pro-inflammatory cytokines (Hansson et al., 2021). These interventions result in a reliable increase of pro-inflammatory cytokines, such as TNF-α and IL-6, in healthy subjects without inducing an actual infection (Balter et al., 2018; Benson et al., 2017; Bollen et al., 2017; Eisenberger et al., 2009; Grigoleit et al., 2011; Kotulla et al., 2018; Kullmann et al., 2014; Moieni et al., 2015b; Schedlowski et al., 2014; Wright et al., 2005). Typical symptoms of sickness-behaviour, such as increase in self-reported depressed mood, anxiety, fatigue, and uneasiness (e.g., Engler et al., 2016; Grigoleit et al., 2011; Kullmann et al., 2014), feelings of social disconnection (Eisenberger et al., 2010; Moieni et al., 2015a) and even lower self-esteem (Kotulla et al., 2018) were repeatedly induced in individuals via endotoxemia. It has been demonstrated that endotoxin injection also leads to alterations in emotion perception. For instance, impaired emotion recognition accuracy (of Reading-the-Mind-in-the-Eyes-Test - RMET) (Balter et al., 2018; Moieni et al., 2015a), negatively biased information processing (Benson et al., 2017; Bollen et al., 2017; Hansson et al., 2021), disturbed reward processing (Harrison et al., 2016) and altered emotion-regulation were shown (Hansson et al., 2021). Those alterations were accompanied by heightened brain activity in areas that are associated with different aspect of neural processing of emotions and motion (for a review, see: Harrison et al., 2016; Lasselin et al., 2018).

Besides inflammation-induction via endotoxemia, there are potentially sterile options, such as physical exercise-induced stress that also induce temporarily pro-inflammatory processes. Particularly intense and prolonged endurance exercise induces a mild pro-inflammatory response characterized by an increase of circulating leukocytes and inflammatory cytokines, such as IL-6 and TNF-alpha, which is followed by an anti-inflammatory counter regulation in the post-exercise period (Peake et al., 2015b). The physiological basis for this exercise-induced immune response is a stress response associated with the release of catecholamines, increased blood flow, a rise in body temperature, ultrastructural tissue trauma, and increased challenges on energy metabolism (Hodgman et al., 2023). The pro-inflammatory exercise response is even more pronounced after acute exercise that is unfamiliar and involves high muscle tension combined with eccentric components (Peake et al., 2005).

Given that both familiar and unfamiliar forms of exercise have been linked to the systemic release of pro-inflammatory cytokines (Suzuki, 2018), it is hypothesized that there exists a reciprocal relationship between neuronal and immunological mechanisms during exercise. This interplay is posited to influence social cognitive processing (Proschinger and Freese, 2019).

On this background, our within-subject design aims to explore the impact of exercise-induced pro-inflammatory responses on the perception and visual exploration of emotions conveyed through body language during social interactions. We employed 45 min downhill treadmill running at 70% VO2max, as an intensive eccentric exercise bout to induce a high level of pro-inflammatory cytokines, leading to a systemic pro-inflammatory status. Two control conditions were selected: a control run level run and a rest condition without physical exercise. Three hours later, participants observed point-light displays (PLDs) of human interactions portraying four emotions (happiness, affection, sadness, and anger). Participants were asked to categorize the depicted emotional content, the emotional intensity of the perceived stimulus as well as their confidence regarding their ratings. We furthermore assessed eye movements of the participants during the whole paradigm as well as their self-reported current mood. We hypothesize that exercise-induced inflammation alters emotion perception with respect to its accuracy, the perceived emotional intensity and the subjective confidence of these ratings as well as the visual exploration behaviour.

## 2 Materials & methods

### 2.1 Participants

Altogether 19 male adults aged 19–33 (mean age: 23.89 years; SD = 3.78 years) with normal or corrected-to-normal vision participated in our study. None of the participants reported any history of psychiatric, neurological, immunological or physical disorders, a current use of psychoactive medication or performance of endurance exercises on more than 4 days a week. Prior to participation, all participants gave written informed consent in compliance with the Declaration of Helsinki. Due to Corona policy all participants had to wear surgical or FFP2 masks. Additionally, they had to be symptom-free and give written confirmation thereof. All participants were either vaccinated against COVID-19 prior to or during participation in this study. Additionally, the state of health of each subject was checked with a blood count before exercise. In case of anomalies, subjects were excluded from participation. The procedure was approved by the local ethics committee of the Department of Psychology and Sports Science of Justus-Liebig-University Giessen. We used the German version of the Emotional Competence Questionnaire (Emotionale-Kompetenz- Fragebogen, EKF; Rindermann, 2009) to assess the participant's ability to recognize and understand one's own emotions; the ability to recognize other's emotions; the ability to regulate and control one's emotions; the ability to express one's emotions (nonverbally and verbally). Normalized standard mean EKF values were in a normal range (90.1–109.9) for all four main domains. Additionally, the German version of the Interpersonal-Reactivity-Index (IRI-SPF, Saarbrückener Persönlichkeitsfragebogen zur Messung von Empathie, V 7.0 Paulus, 2009) was used to estimate the participant's empathic competence within four emotional and cognitive empathic factors. The *empathy scores* were all in a normal range (mean 43.77, SEM 1.30). Participants' scores for EKF items and IRI-SPF did not differ between the experimental conditions.

### 2.2 Experimental procedure

**Fig. 1:**
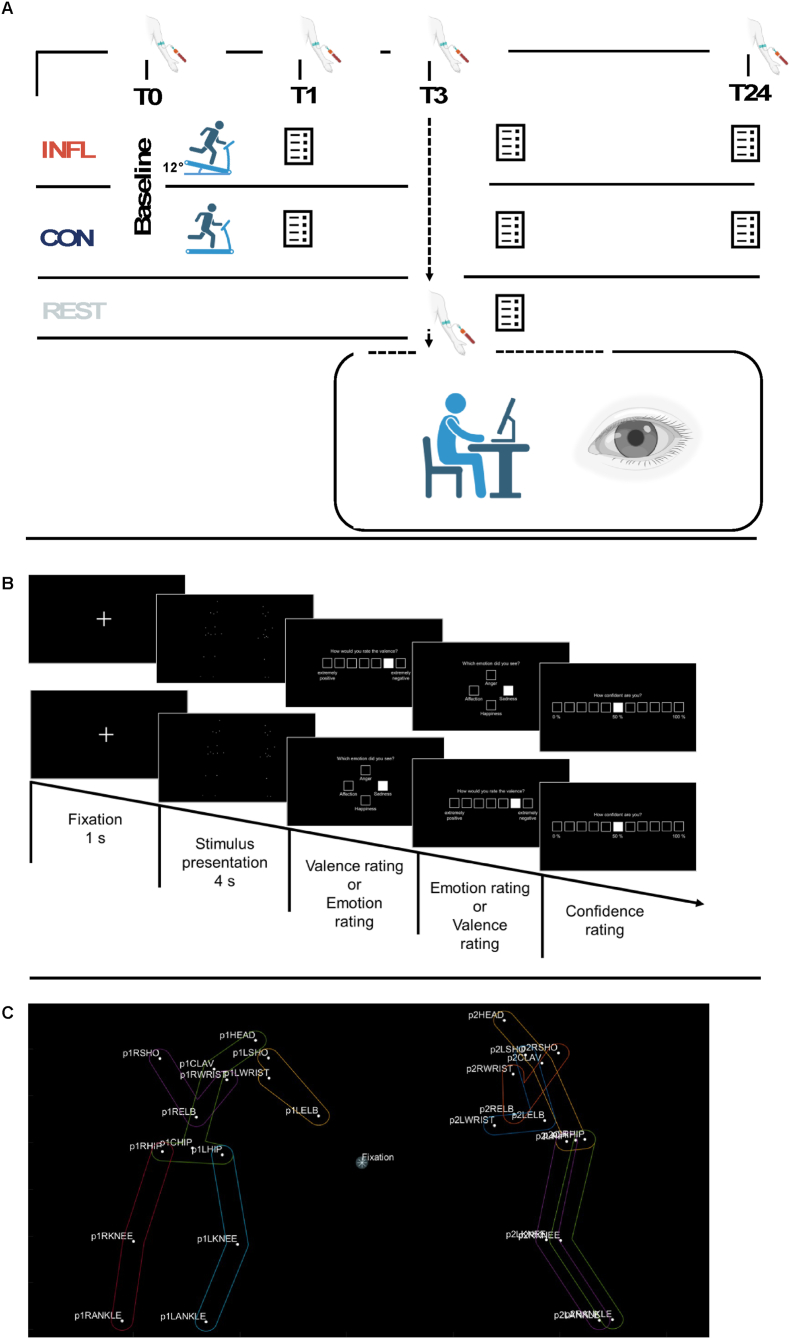
*Experimental Procedure and Timeline*. A) Time-schedule of experimental conditions, blood-sampling, experiment and questionnaires. B) Temporal structure of two trials, each trial started with a fixation phase (1s) followed by the stimulus sequence (4s). Subsequent to the stimulus presentation, an emotion and valence rating (upper row) were presented. In half of the trials, the ratings were inverted (lower row). These ratings were followed by a confidence rating. C) Anatomical landmarks and predefined AOIs for eye-tracking analysis.

The study was conducted as a within-subject cross-over design. Participants visited our laboratories on four different days, with at least one week between each testing day: Pre-test, downhill running (INFL), control running (CON) and rest (REST). Order of INFL and CON treadmill running were randomized across participants, REST was always scheduled for the last session. This was necessary because blood cells derived from REST blood sampling had to be directly used in another experiment in which these cells were incubated with the different sera of the two exercise conditions (Gebhardt et al., 2024). An overview of general procedure is shown in Fig. 1 A.

On the first day, each individual's anthropometric data (height, age, weight), pulmonary function (vital capacity, forced expiratory volume), electrocardiogram and blood pressure at rest, as well as a body fat analysis (via bioelectrical impedance analysis) were assessed by a physician. After that, participants were tested to obtain their individual maximal oxygen uptake (VO_2max_) using a step protocol on the treadmill starting at 8 km/h with a step height of 2 km/h and step length of 2 min until subjective exhaustion. VO_2_ (via spiroergometry), heart rate (via chest strap) and the subjective exhaustion (via BORG-scale) were assessed at every step. VO_2max_ was used to determine the individual's running speed for INFL and CON protocols (Gebhardt et al., 2024).

Downhill treadmill running (12% decline) with a VO_2_ corresponding to 70% of the individual's VO_2max_ was selected as an inflammatory stimulus (INFL). This type of eccentric exercise containing high intensity and a long duration is known to induce a reliable and notable increase in circulating pro-inflammatory cytokine levels (e.g., IL-6, TNF-α, CRP, MCP1) without inducing significant effects on sickness symptoms such as fever (Peake et al., 2005; Steensberg et al., 2002). As a control condition for possible effects related to running itself, such as cardiopulmonary stimulation etc., control treadmill running with the same intensity at 1% incline was selected (CON).

Durations of both running protocols were set to 45 min unless the participants were forced to quit earlier due to exhaustion or other reasons. Participants had to run for at least 30 min to be included into data analysis. Only one of the participants had to stop the run after 35 min in the downhill condition. Both runs were completed on a treadmill by h/p/cosmos (h/p/cosmos quasar med 4.0, h/p/cosmos Sports & Medical GmbH, Nussdorf, Traunstein, Germany) and began with a short warm-up of 10 min in which VO_2_ was regulated to match the 70% VO_2max_ via adjustments in velocity. For each condition and participant, the sessions have been scheduled between 8 a.m. and 12 p.m. to account for potential variations in the blood concentration of inflammatory markers that could occur in the afternoon (Nakao, 2014).

### 2.3 Assessment of inflammatory status

Venous blood samples were collected at four points in time in each experimental condition (INFL, CON) (prior to exercise = T0; immediately after exercise = T1; 3 h after exercise = T3; 24 h after exercise = T24) in vacutainers. The level prior to exercise (T0) served as baseline for the two exercise conditions (see Fig. 1. A). Plasma vacutainers were anticoagulated with EDTA. They vacutainers were centrifuged at 2500×*g* for 10 min at 4 °C immediately after sampling, while serum samples had clotted for 30 min before centrifugation. Samples were separated into aliquots and stored in Eppendorf tubes at −80 °C until analysis. IL-6, TNF-α, CRP, MCP1 levels as well as Myoglobin (Mb) as a marker of muscle damage were determined by Luminex Multiplex Assay (Luminex LX200, Biotechne, Minneaplois, USA).

### 2.4 Assessment of mood state

To describe participants’ current mood state at each testing day, a short version of the German mental state scales (Befindlichkeitsskalen – BFS, Abele-Brehm and Brehm, 1986) was used. Since negative mood and anxiety were reliably induced by endotoxemia before (e.g., Engler et al., 2016; Grigoleit et al., 2011; Kullmann et al., 2014), Anger, Positive Mood, Calm, Arousal and Depressed were selected to describe mood-related items due to their relevance for the connection between mood and inflammation. The BFS contains 21 5-point-scaled items ranging from 1 (never) to 5 (always).

### 2.5 Emotion recognition experiment

Three hours after exercise**,** participants were given instructions and acquainted with the task. They subsequently performed three example trials that were not included in the main experiment. In the experiment, each video was presented once, resulting in a series of 48 sequences. Video sequences were displayed in an intra- and interindividually randomized order on a 19-inch screen (display resolution: 1280x720, refresh rate 60 Hz). The distance between each test person's eyes and the screen was approximately 65 cm. After completing a calibration and validation procedure for the eye tracker, each trial started with a fixation phase (1 s) followed by a stimulus sequence (4 s) and three behavioural ratings. The ratings consisted of: 1.) an emotional valence rating, rating the video sequences on a 7-point Likert scale from very negative (−3) to very positive (+3). 2.) Sorting emotions into one of the following categories: happiness, affection, sadness, or anger. 3.) Confidence ratings on an 11-point Likert scale ranging from 0% (−5) to 100% (+5). The order of emotional valence ratings and sorting emotions to categories were alternating to avoid order effects, confidence ratings were always provided last. This procedure is visualized in Fig. 1 B.

To control for potential influences of the inflammatory state on cognitive functions, such as working memory, participants engaged in a spatial span task. This task required them to replicate the location of an object displayed on a computer screen. At the initial level, participants were asked to replicate one location. With each subsequent level, the task's difficulty increased by requiring the replication of an additional location. The highest level at which a participant accurately replicated all locations was assessed and utilized as a control parameter.

### 2.6 Stimuli

The observed stimuli were selected from a larger motion-capture data set from Bachmann et al. (2020). Eight pairs of non-professional actors were instructed to perform an interaction portraying one out of four emotional scenes depicting either happiness, affection, sadness, or anger. To ensure an emotion-congruent behavioural pattern, actors were given a script of emotional situations and directed specifically to perform the same emotion. They were instructed to express their emotions intuitively within the context of the given situation, thereby limiting restrictions and enhancing the variability of expression (Bachmann et al., 2020). Interactions were recorded with an optical motion capture system (Vicon Motion Systems, Oxford, England) operating at 100 Hz. MATLAB software (Mathworks, Natick, MA) was used to create video files of 4-s sequences from the original coordinate 3D (C3D). In each video, 15 markers per person were then plotted as white spheres on a black background to present a standard PLD (point-light display) model.

The final stimulus selection was based on prior validation of emotion category (anger, sadness, affection, happiness) and perceived valence from 24 participants who did not take part in the present experiment. Valence was judged on an 11-point scale ranging from −5 (extremely negative) to +5 (extremely positive). There were two validation criteria: 1.) At least 50% of the participants had to recognize the displayed emotion; 2.) The second-highest emotion rating should not exceed 25%. This allowed us to identify and exclude ambiguous scenes in which a specific emotion could not be recognized reliably and those which were overly easy to recognize, avoiding both floor and ceiling effects. After validation, 12 stimuli that met both criteria were selected randomly for each emotion category, which resulted in a set of 48 (4 emotions × 12 scenes) stimuli.

### 2.7 Eye tracking

During the whole experiment, a video-based pupil- and corneal reflection Tobii Nano Pro eye tracker (Tobii AB, Danderyd, Sweden) was used to track participant's gaze behavior during stimulus presentation at a sampling rate of 60 Hz and mounted on the bottom of the screen. According to the manufacturer, the eye tracker operates with a precision of 0.1° RMS and an accuracy of 0.3° in optimal conditions with a total system latency of 1 frame (17 ms). To determine fixations, we identified events in between saccades with a minimum event duration of 100 ms and a saccade velocity threshold of 30 deg/s (de Haas et al., 2019). Note that this definition of fixation also encompasses pursuit phenomena, as it can be assumed that with dynamic stimuli, the eyes wander along, and this is equally essential for a corresponding fixation.

Within all 48 PLD stimuli, for each actor separately and within every frame of the video, five areas of interests (AOI) based on the 2D dot coordinates of the markers were created using Matlab (2022b) with the corresponding labels: right arm, left arm, trunk, right leg and left leg. In order to determine which fixation or pursuit events fell into which area, two criteria were used. 1.) All distances between the event and all markers were calculated to find the minimum distance across all relevant frames. 2.) Additionally, this resulting minimum distance had to be below a threshold of 50 pixels to count as a fixation of the area, which served as a tolerance margin to account for oculomotor drift and potential accuracy limits (see Fig. 1 C).

### 2.8 Data analysis and statistics

Prior to statistical analysis, several pre-processing steps of the data were carried out. First, the current mood state was determined by summing up the selected items of the BFS after each exercise condition. Higher values in the sum score reflect increasing negative mood and anxiety. To determine emotion recognition accuracy in the EBL paradigm, the percentage of correct category ratings of each emotion was calculated by comparing the rating of a given stimuli to the actual validated emotional category. To determine intensity perception of the PLDs, absolute valence values were determined to be able to make an inference about how intense a stimulus was perceived. Thus, higher intensity can therefore be interpreted as more negative or more positive, depending on the category. Confidence ratings were calculated to gather information about the subjective impression of the participants' confidence regarding the given rating. For the sensitivity measurement d’, we calculated the proportion of hits (HR) (identified and displayed emotion are consistent) and false alarms (FAR) (identified and displayed emotion are inconsistent). Proportions of 0 were replaced with 0.5/N_p/a_:

$$d^{\prime }=z\left(\text{HR}\right)\;–\;z\left(\text{FAR}\right)$$

Sensitivity reflects the observer's ability to discriminate whether the ‘signal’ (here, the respective emotional category) is present or not. High d' values indicate a good discrimination ability for the respective emotional category, i.e., the category can be distinguished from the other ones. Each participant's d’ values were calculated as the mean for each emotion, separately for all three conditions.

### 2.9 Exercise-induced inflammation

To ascertain whether a given exercise-intervention leads to an inflammatory response, we tested whether there was a condition-dependent change of cytokine concentrations from T0 to T1 to T3 to T24. Therefore, we conducted a 2 (condition: INFL vs. CON) x 4 (time-point: T0, T1, T3, T24) repeated-measures ANOVA for IL-6, TNF-α, CRP and MCP1 as markers for inflammation, as well as for myoglobin (Mb) as a marker for muscle damage. It has to be noted that missing values exist for each parameter. This is because the measured values lie outside the normal curve and, therefore, cannot be determined.

### 2.10 Influence of exercise-induced inflammation on mood

To determine possible influences of exercise-induced inflammation on current mood state of participants, we performed a one-way ANOVA with a three-level factor exercise condition (INFL, CON, REST).

Influence of exercise-induced inflammation on emotion recognition, intensity perception, confidence and sensitivity.

To ensure adequate recognizability of the displayed emotions, emotion recognition accuracy was tested against chance (25 %) for all conditions via Wilcoxon signed-ranks test.

To determine the possible influence of exercise-induced inflammation on emotion recognition accuracy, intensity perception as well as perceived confidence, for each parameter a 3 (condition: INFL, CON, REST) x 4 (emotion category: happiness, affection, anger, sadness) repeated-measures ANOVA was conducted.

To investigate whether inflammation induced changes in perceptual sensitivity, we calculated a 3 (condition: INFL, CON, REST) x 4 (emotion category: happiness, affection, anger, sadness) repeated-measures ANOVAs using d’.

To assess the impact of exercise-induced inflammation on working memory performance, we conducted a one-way ANOVA with the exercise condition as a three-level factor (INFL, CON, REST).

### 2.11 Influence of exercise-induced inflammation on eye movements

To determine the possible influence of exercise-induced inflammation on gaze behavior, we quantified different eye movement measurements (Suslow et al., 2020).). Visual scanning was described by the numbers of fixations (NF) and average glance duration (AGD). The so-called initial attention was measured by the latency of the first fixation (First Fixation Latency = FFL) as well as the duration (First Fixation Duration = FFD). The maintenance of attention was evaluated by calculating dwell time (DT). For more detail on the calculation see Table 1.

**Table 1:** Description of gaze parameters.

Parameter	Abbr.	Short description
Number of Fixations	NF	Number of fixations for each trial averaged across emotions
Average Glance Duration	AGD	Average amount of time each subject's gaze stays within boundaries of the AOIs (calculated by dividing fixation time by fixation frequency)
First Fixation Duration	FFD	Duration of first fixation in ms
First Fixation Latency	FFL	Time between stimulus onset and first fixation in ms
Dwell Time	DT	Sum of durations from all fixations that hit AOI in ms. Calculated for each AOI and each trial and then averaged for each subject

For simplicity we refer to both pursuit and fixation events as ‘fixations’.

For all parameters of gaze behavior, we calculated separate 3 (condition: INFL, CON, REST) x 4 (emotion category: happiness, affection, anger, sadness) repeated-measures ANOVAs.

For each conducted repeated-measures ANOVA, Mauchly's test of sphericity was conducted. Violations were treated by using Greenhouse-Geisser (ε < 0.75) correction (Girden, 1992). If repeated-measures ANOVA displays significant effects, post hoc pairwise comparisons were calculated and Bonferroni-corrected. For all pre-processing steps and statistical tests, Matlab R2022b and JASP 0.15.0.0 were used. Alpha significance levels were set to 0.05 for all statistical tests.

## 3 Results

### 3.1 Muscle damage and inflammation after exercise

**Fig. 2:**
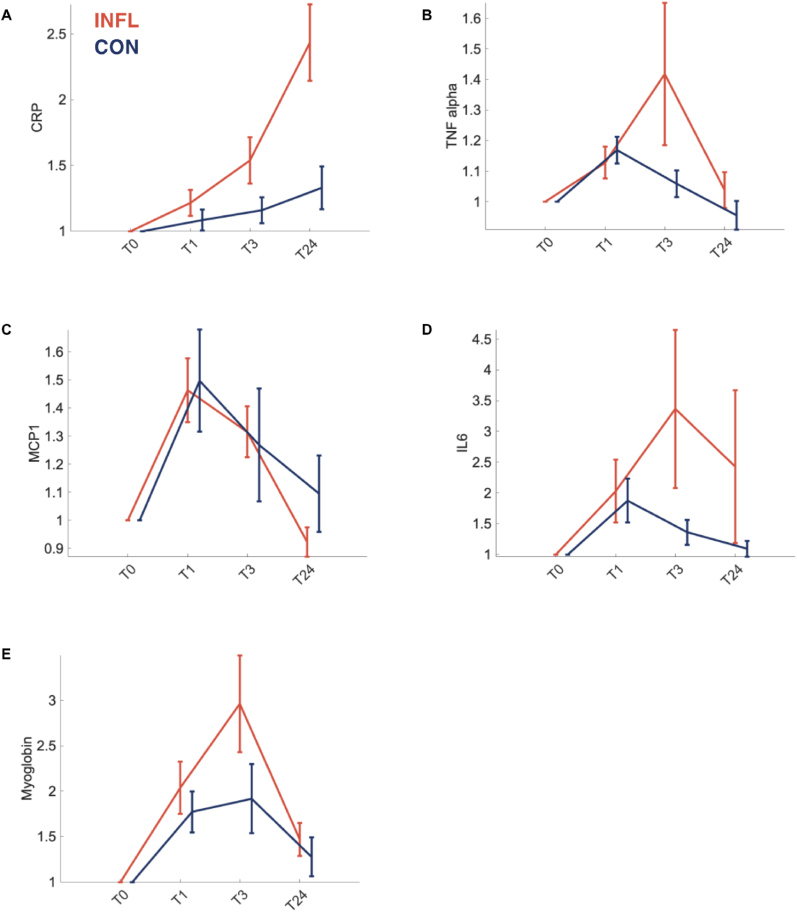
*Concentration of various cytokines (B, C, D) and plasma proteins (A, E)* prior to exercise = T0; immediately after exercise = T1; 3 h after exercise = T3; 24 h after exercise = T24; downhill-running (INFL/red) or control-running (CON/blue). The depicted data are the means and the SEMs of biomarker increases relative to T0.

Using a 2 × 4 repeated-measures ANOVA, we investigated whether the levels of IL-6, TNF-α, CRP, MCP1 and Mb were affected differentially by the different exercise conditions in order to verify that INFL leads to a significant systemic inflammation which is more pronounced than the inflammatory response after CON (see Fig. 2).

Our results revealed an interaction effect of experimental condition x time point for IL-6 (see Fig. 2 D) concentrations (F(3,16) = 7.535, p < 0.05, η^2^ = 0.079). Bonferroni-corrected post hoc analyses showed that blood concentration levels of IL-6 were significantly higher for INFL T3 compared to INFL T0 (p < 0.001) and CON T24 (p < 0.05). We found no main effect of condition for IL-6.

For CRP (see Fig. 2 A), a main effect of condition was found (F(1,16) = 4.558, p < 0.05, η^2^ = 0.05). Post hoc analysis revealed higher blood concentration of CRP for INFL compared to CON (p < 0.05). Furthermore, Greenhouse-Geisser corrected values revealed an interaction effect of experimental condition x time point (F(1.73,16) = 6.650, p < 0.01, η^2^ = 0.074). Bonferroni-corrected post hoc analyses highlights differences between INFL T0, T1, T3 and CON T0, T1, T3, T24 compared to INFL T24 (p < 0.001).

Results for MCP1 (see Fig. 2 C) revealed an interaction effect between condition x time point (F(3,16) = 23.643, p < 0.05, η^2^ = 0.034) but no effect of experimental condition. Regarding the interaction effect, INFL T1 showed a higher MCP1 concentration compared to CON T0, T3 and T24 as well as to INFL T0, T24 (all p < 0.01). Likewise, CON T1 was higher compared to CON T0 and CON T24 (p < 0.001) as well as to INFL T24 (p < 0.01). Lastly, INFL T3 MCP1 concentration was higher compared to both T0 and T24 of both conditions (p < 0.01).

Mb concentrations (see Fig. 2 B) were measured as a possible inflammation-driving factor under INFL. For Mb concentrations, a significant main effect for condition (F(1,18) = 5.214, p < 0.05, η^2^ = 0.042) was found. However, there was no interaction effect. Post hoc analysis showed that the Mb concentrations were higher for INFL compared to CON (p < 0.05). Furthermore, paired sample t-tests indicated a higher Mb concentration for INFL T3 compared to CON T3 (p < 0.05).

There was no main of experimental condition and no interaction between condition and time point for TNF-α.

### 3.2 Mood state

Regarding the mood state (Anger, Positive Mood, Calm, Arousal, Depressed), we found no differences of any of the investigated mood scales between the different experimental conditions (all p > 0.05).

### 3.3 Influence of exercise-induced inflammation on emotion recognition

**Fig. 3:**
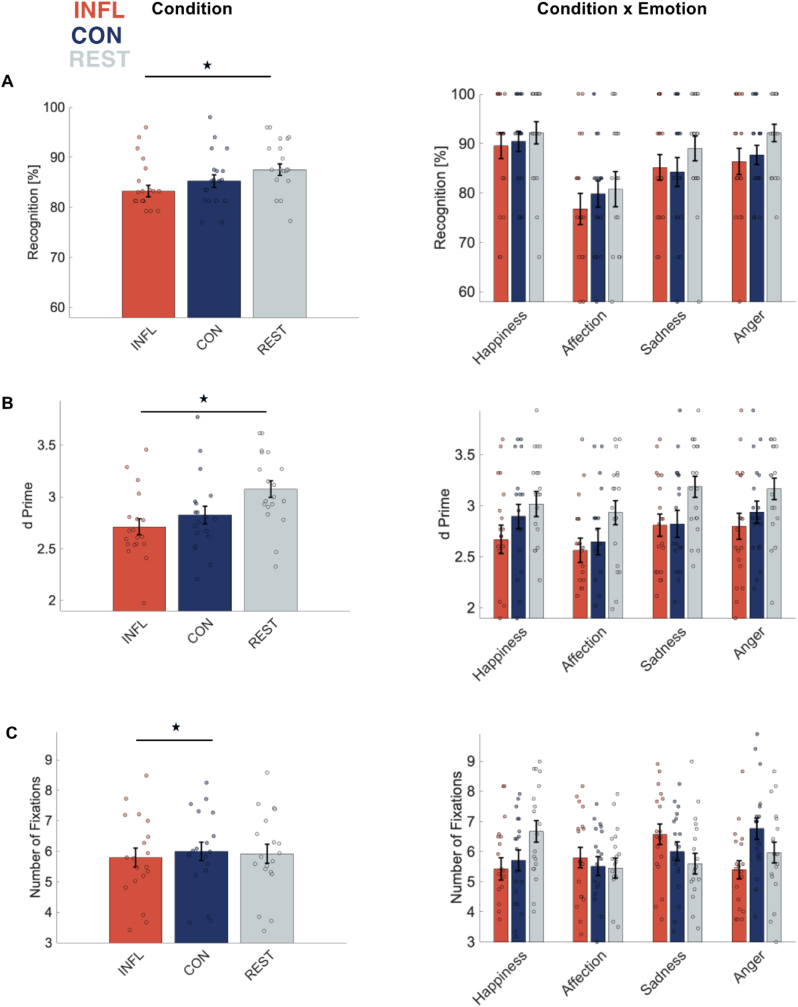
*Influence of experimental condition on recognition performance and gaze behavior.* A) Emotion recognition rates of interactions for all emotion categories (i.e, happiness, affection, sadness, anger). B) Average sensitivity (d’) and C) Number of Fixations by condition and emotional category. Bars and error bars show means and SEMs. Significance level between conditions is indicated by asterisk. (⋆ p < 0.05).

Overall, emotion category ratings given here show a high consistency with the ratings from the validation study (see Fig. 3 A). The Wilcoxon signed-rank test revealed that sequences of each emotional category were classified significantly above chance level (all p < 0.001) and thus ensuring a high degree of recognizability.

Using a 2 × 4 repeated-measures ANOVA, we investigated whether the recognition of emotional interactions was affected by exercise-induced inflammation. Results revealed a main effect of experimental condition on recognition performance (F(1.790,18) = 4.465, p < 0.05, η^2^ = 0.023). Bonferroni-corrected post hoc analyses showed that recognition rates for REST were significantly higher compared to INFL (p < 0.05) but not to CON. Furthermore, a main effect of emotion category was found (F(2.279,18) = 5.823, p < 0.05, η^2^ = 0.147) with post hoc analyses indicating that affection scenes were accompanied by significantly lower recognition rates compared to happiness and anger scenes (p < 0.05) while other pairwise comparisons revealed no significant differences (all p > 0.05). We found no interaction effect between condition and emotion (see Fig. 3 A).

Regarding intensity ratings, we found no main effect of condition as well as no interactions between condition and emotion category (all p > 0.05). However, a main effect was present regarding emotion category (F(2.153,18) = 44.559, p < 0.001, η^2^ = 0.483) with post hoc analysis showing that interactions portraying happiness were perceived more intense compared to the all other emotions (all p < 0.001). Further, affection stimuli were perceived more intense compared to sadness stimuli (p < 0.005).

The same main effect of emotion category was present for confidence ratings (F(3,18) = 15.860, p < 0.001, η^2^ = 0.253) with participants confidence in their rating being higher for happy interactions compared to all other emotional interactions (all p < 0.001). No further effects were revealed regarding the perceived confidence.

To investigate how inflammation influences participants' discrimination abilities, we calculated d’ (see Fig. 3 B). We found a main effect of condition, (F(2,18) = 5.784, p < 0.001, η^2^ = 0.093) with increasing sensitivity for REST compared to INFL (p < 0.05). The present data revealed no main effect of emotion category and no interaction between condition and emotion category for d’.

In the control analysis, we found no differences in working memory performance across the experimental conditions (F(2,18) = 1.441, p > 0.05).

### 3.4 Influence of exercise-induced inflammation on eye movements

Using 2 × 4 repeated measures ANOVAs for all eye movement related parameters, results showed that the exercise induced inflammation status of participants influenced the number of fixations (NF) (F(2,18) = 3.947, p < 0.05, η^2^ = 0.011) (see Fig. 3 C). Post hoc comparisons revealed more NFs for INFL compared to CON (p < 0.05), as displayed in Fig. 3C. Additionally, a significant main effect of emotion occurred (F(3,18) = 4.395, p < 0.01, η^2^ = 0.058) with less fixations when viewing affectionate interactions compared to both negative emotion displays (all p < 0.05). Greenhouse-Geisser-corrected interactions between emotion and condition (F(3.951,18) = 15.765, p < 0.001, η^2^ = 0.302) showed that happy interactions were less fixated in INFL compared to REST, whereas they were more often fixated in REST compared to CON (all p < 0.05). Sad interactions under INFL were more fixated compared to REST. Furthermore, angry interactions in INFL were less fixated than angry interactions in CON (all p < 0.05). Under INFL, happy and affectionate as well as angry interactions were less often fixated than sad interactions (all p < 0.05). For CON, both positive interactions were less often fixated than angry interactions (all p < 0.05). In the REST condition, happy interactions were significantly more often fixated than affectionate and sad interactions (all p < 0.05).

In addition to the number of fixations, we examined the average glance duration (AGD). Greenhouse-Geisser-corrected values indicated a main effect of emotion category (F(1.944,18) = 3.782, p < 0.05, η^2^ = 0.06) with post hoc analysis showing longer AGDs for affectionate compared to sad and angry interactions (p < 0.05). There was no significant effect of experimental condition regarding average glance duration. However, Greenhouse-Geisser-corrected values indicated an interaction effect (F(3.142,18) = 6.680, p < 0.001, η^2^ = 0.154) between condition and emotion category. Post hoc comparisons revealed longer AGDs for happiness stimuli in INFL compared to REST and anger stimuli in INFL compared to CON (all p < 0.05). Sad interactions were accompanied by shorter AGDs in INFL compared to REST (p < 0.05). In INFL, observing happy interactions led to longer AGDs than sad interactions (p < 0.05). In CON, anger scenes led to shorter AGDs compared to happy and affectionate scenes (all p < 0.05).

Focusing on the effect of emotion category regarding the initial attention, we found a main effect concerning the duration of the first fixation duration (FFD) (F(3,18) = 2.828, p < 0.05, η^2^ = 0.05) revealing longer FFDs when observing affectionate compared to sad scenes (p < 0.05). We found no effects of experimental condition regarding the duration of the first fixation. However, Greenhouse-Geisser corrections revealed a significant interaction between emotion and condition (F(3.767, 18) = 5.365, p < 0.001, η^2^ = 0.128). Post hoc comparisons further elucidated that initial fixations in anger scenes were notably longer in CON than INFL (p < 0.05). Also, angry scenes in CON elicited longer initial fixations than in INFL (p < 0.05).

For the latency of the first fixation (FFL), we did not find a main effect of experimental condition or emotion category. However, there was an interaction between experimental condition and emotion category (F(6,18) = 2.384, p < 0.05, η^2^ = 0.06). All post hoc comparisons did not reveal statistically significant differences concerning the latency of the first fixation.

Attention maintenance of participants depicted through dwell time (DT) showed no main effect of either experimental condition or emotion category. Though, an interaction effect (F(2.995,18) = 7.206, p < 0.001, η^2^ = 0.151) with Greenhouse-Geisser-corrected values indicating longer DTs for affectionate interactions in REST compared to happy and sad interactions in the same condition (all p < 0.05). Overall, observing affectionate interactions was accompanied by shorter DTs for REST compared to INFL and CON (both p < 0.05).

## 4 Discussion

An increasing number of studies (e.g. Eisenberger et al., 2009; 2010; Moieni et al., 2015a, Moieni et al., 2015b) have explored the impact of systemic inflammatory conditions on emotion perception and theory of mind. This study employed a within-subject design in a cohort of young, healthy males to explore the potential impact of exercise-induced sterile inflammation on the recognition and visual scanning behavior of emotions conveyed through body language during emotional interactions. Specifically, we induced an inflammation-like state using a downhill running exercise model. This is evidenced by elevated muscle damage markers and an increase in pro-inflammatory cytokines and serum proteins. The eccentric downhill exercise condition resulted in greater elevations of measured cytokines (IL6, CRP, MCP-1) and markers for muscle damage (Mb) compared to the control running condition (Bruunsgaard et al., 1997; Peake et al., 2005; Pedersen and Febbraio, 2008; Steensberg et al., 2002). Notably, we observed significant increases in IL-6 and MCP1 between T0 and T3 only in the downhill running condition, while CRP levels differed between both conditions only at T24. There were no significant differences between the conditions regarding TNF-α levels. Particularly at T3, the time point of the behavioral experiment, myoglobin concentrations substantially increased in the downhill run compared to the control run, indicating the presence of a noticeable muscle damage as a driver of inflammation.

Regarding emotion perception, we demonstrated significantly decreased recognition rates of the emotional state of two interacting PLD agents for 3 h after downhill running compared to the rest condition, but no such effect was observed for 3 h after control running compared to rest. The experimental condition had no effect on the perceived emotional intensity or the perceived subjective confidence of the given ratings. Regarding participants' discrimination abilities, our findings indicate that the inflammation condition is accompanied by a reduced ability to differentiate between emotion categories compared to the rest condition. We also observed that the inflammatory condition is associated with changes in participants' visual scanning behavior, potentially causing them to exhibit more fixations on sad stimuli, in contrast to the control condition, where they exhibited more fixations while observing happy stimuli. In the next sections, our results are discussed in more detail.

### 4.1 Strenuous exercise as an experimental model to induce inflammation

An important feature of the present study is that we used an exercise model to induce a pro-inflammatory condition. It has been repeatedly shown that strenuous bouts of endurance exercise and particularly exercise with eccentric components induce a pro-inflammatory response (Peake et al., 2017b). Indicators of inflammation, for example, are mild increases in systemic IL-6 and TNF-α in the post exercise period, which occur immediately and are often present for up to 48 h (Paulsen et al., 2012). While this is fundamentally part of the exercise-induced immune response, the eccentric load of the downhill run adds muscle damage, resulting in a more pronounced sterile inflammatory process. In the present study, this component of downhill exercise is indicated by elevated myoglobin levels as a marker of muscle damage, as well as more increased IL-6, CRP and MCP1 levels compared to the control condition (Peake et al., 2005). Yet, regarding TNF-α only a trend was visible in our data.

Notably, the exercise intervention employed in this study elicited a much smaller inflammatory response in comparison to the endotoxin manipulations utilized in related research (Eisenberger et al., 2009, 2010; Engler et al., 2016; Grigoleit et al., 2011; Kotulla et al., 2018; Kullmann et al., 2014; Moieni et al., 2015b, Moieni et al., 2015a; Wright et al., 2005; Lasselin et al., 2018). For instance, Moieni et al. (2015) employed endotoxin as an inflammatory challenge to investigate inflammation-induced alterations in a theory of mind task. The endotoxin injection resulted in a 100-fold increase in IL-6 and TNF-α levels, accompanied by various mood-worsening effects, whereas the current exercise intervention led to an average increase of only 3–4 fold of IL-6 compared to baseline.

However, inducing smaller increases in inflammation may offer advantages for studies focusing on the social cognitive consequences of low-grade inflammation (cf. Balter et al., 2018) as the level of immune activation demonstrated in this study more closely resembles the low-grade inflammatory levels observed in individuals with depression, as well as in medical conditions like diabetes. For example, IL-6 levels in people with type-2 diabetes or depression often range between 2 and 10 pg/ml (Kadoglou et al., 2007). Thus, the current model appears to provide a more accurate representation of the inflammatory state in the mentioned medical conditions compared to many endotoxin studies. The relatively modest increases in inflammatory markers observed in this study can be attributed to the nature of sterile inflammation, which elicits milder levels of inflammatory cytokines compared to inflammation induced by bacteria or bacterial components. This approach minimizes potential confounding side effects, such as sickness symptoms or significant mood deterioration, previously reported in other studies. (e.g., Dowlati et al., 2010; Maes et al., 1995). Furthermore, the present method offers advantages, including sterility, by avoiding skin and tissue injury, eliminating infection risks, and circumventing administration challenges, as noted in prior research (Shek and Shephard, 1998; Wright et al., 2005). Another benefit of our exercise-induced inflammatory treatment is its adaptability to each participant's baseline fitness level, making the approach suitable for individuals with varying physical activity levels.

### 4.2 The influence of inflammatory processes on emotion perception

Regarding emotion perception, we revealed inflammation-driven changes in emotional state recognition of two interacting PLD agents. Specifically, in a pro-inflammatory state induced by downhill running, individuals showed reduced emotional content recognition during observed interactions irrespective of the displayed emotion. Moreover, their sensitivity to emotion-specific cues, quantified by d', declined.

The present findings not only reinforce but also enrich the existing body of literature, which has consistently demonstrated that the injection of endotoxins and the ensuing inflammatory response result in significant alterations in emotion perception. This phenomenon has been explored from various angles in prior research. On one front, studies have unveiled a proclivity for inflammation to engender a perceptual bias skewed towards negativity (Benson et al., 2017; Bollen et al., 2017). Simultaneously, other investigations showed that endotoxin-induced inflammation appears to impair one's capacity to interpret emotions portrayed through facial expressions, as assessed by instruments like the Reading-the-Mind-in-the-Eyes-Test (Balter et al., 2018; Moieni et al., 2015). Collectively, these findings, along with those of the current study, suggest that inflammation detrimentally affects the precise recognition of emotional states in others, regardless of the emotion's valence. Furthermore, Muscatell et al. (2016) demonstrated the influence of endotoxin-induced inflammation on neural activity within regions integral to mentalizing, the process of attending to and comprehending others' thoughts and feelings. Notably, individuals subjected to the endotoxin condition exhibited heightened activity in the dorsomedial prefrontal cortex, a pivotal node in the mentalizing network, when exposed to both negative and positive social feedback, in contrast to those in the placebo condition. Consequently, it becomes evident that inflammation exerts a discernible impact on the brain's responsiveness to social and affective stimuli.

In contrast to the frequently reported findings in related literature, our study did not observe an increase in aroused, depressed, or angry mood following exercise-induced inflammation at both the 3-h and 24-h time points after downhill running. This contrasts with several studies that induced inflammatory status via endotoxemia, which showed an increase in self-reported depressed mood, anxiety, fatigue, uneasiness (e.g., Engler et al., 2016; Grigoleit et al., 2011; Kullmann et al., 2014), feelings of social disconnection (e.g., Eisenberger et al., 2010; Moieni et al., 2015a), as well as low self-esteem (Kotulla et al., 2018). As previously mentioned, the exercise intervention used in our study elicited a smaller inflammatory response compared to endotoxin manipulations, which may explain the absence of depression-like mood alterations in our findings. A further noteworthy point concerning this case is raised by studies of Eisenberger et al. (2009) and Moieni et al. (2015b). They found no differences in mood changes following endotoxins for men but for women independently from cytokine elevations, indicating different hormonal contributions to cytokine induced depressed mood changes. It is important to highlight this aspect to contextualize our results, which were collected from a male cohort.

### 4.3 The influence of inflammatory processes on eye movements

Gaze patterns are recognized as indicators of various conscious and unconscious cognitive processes, including attention allocation, initial vigilance, gaze direction, and focus of attention, as well as attentional engagement and maintenance (Suslow et al., 2020). These processes play a crucial role in regulating emotional responses by shaping initial attention and subsequent processing filters (Todd et al., 2012). However, prior research on the impact of low-grade inflammation on emotion perception has not yet explored eye movement behavior. In our study, we used eye tracking parameters to investigate participants' initial attention, attention maintenance, and visual scanning under different inflammatory conditions. Specifically, we found an effect of experimentally induced inflammation on eye movements, focusing on the number of fixations made. Our findings revealed that the number of fixations increased nearly linearly from the inflammatory (INFL) to the control (CON) and rest (REST) condition when participants observed happy interactions. Conversely, for sad interactions, the number of fixations decreased from INFL to CON to REST. This suggests that participants tended to explore sad stimuli more when experiencing a more pronounced inflammation, and to explore happy stimuli more when this is not the case. Interestingly, this eye scanning behavior aligns with patterns observed in unmedicated depressed patients, who tend to show fewer fixations on positive stimuli and more on negative scenes compared to healthy controls (Suslow et al., 2020). Affective cognition, such as negative biases, is a core aspect of depression (Elliott et al., 2011) and is linked to depression risk, exacerbation, and symptom maintenance. This similarity in gaze behavior suggests a potential indicator of an early depression-like behavior in the presence of acute inflammation.

However, it is important to note that the observed differences in gaze behavior cannot easily explain the present changes in perceptual performance during inflammation. Under inflammation, there was a reduction in emotion recognition for all emotions, while gaze behavior shifted towards exploring sad stimuli more. Interestingly, there was no sensitivity difference for recognizing sad stimuli between the INFL and CON condition, suggesting that the increased tendency to explore sad stimuli may have compensated the detrimental effect of inflammation for these stimuli. Futures studies could further investigate the potential role of affective attention in mediating effects of inflammation on emotion recognition, including a possible role of microsaccades.

### 4.4 Limitations

Firstly, we have to admit that the question of whether the observed effects are solely related to emotion perception or if participants would perform poorly on any perceptual or cognitive task under inflammatory conditions remains partially unanswered. To address this, future studies could incorporate a perceptual control task, such as assessing speed discrimination of randomized point-light displays. However, we showed in a control task that inflammation did not influence working memory capacity. This suggests that inflammatory conditions may primarily impact emotion perception rather than cognition itself. Furthermore, our work indicates that modulations of gaze behavior by inflammation strongly interact with emotional stimulus content, underscoring their affect-specific nature. In this vein, a literature review conducted by Bollen et al. (2017) did not find compelling evidence linking acute experimental inflammation to changes in attention, executive functioning, or consistent patterns in memory functions. In contrast, studies examining social and emotional processing consistently showed that these processes are more likely to be influenced by inflammation.

Second, we acknowledge that the effects of chronic inflammation, as seen in clinical populations, could differ substantially in both nature and magnitude from those observed in our current experimental paradigm (cf. Bollen et al., 2017). Therefore, further research involving individuals with chronic inflammatory conditions is essential to gain a clearer understanding of how chronic inflammation affects emotion perception.

Third, it is important to note that our study only included male participants to mitigate the influence of menstrual cycle fluctuations and enhance controllability. However, existing literature has demonstrated differences between male and female participants concerning emotion perception (Alaerts et al., 2011) and sensitivity to inflammation-induced changes (Engler et al., 2016; Lasselin et al., 2018; Moieni et al., 2015b; Wegner et al., 2017). Therefore, it is highly desirable to conduct studies that encompass both sexes to account for possible sex-specific differences.

A fourth critical concern relates to the current emotion recognition paradigm, which primarily consisted of stimuli with minimal ambiguity. As a result, the overall recognition rates were high, and the variation between conditions was relatively small, making it challenging to detect robust effects. For future investigations, it would be beneficial to incorporate more difficult and ambiguous stimuli to enhance the potential for significant findings.

Another aspect worth considering is the sequence of our experimental conditions, where the rest condition was consistently placed last due to methodological constraints, and the control condition resulted in slightly elevated cytokine levels. Despite this limitation, we want to emphasize that only the contrasts between the downhill running condition and the rest condition yielded significant results. This suggests that the observed perceptual differences are likely attributed to the inflammation condition.

## 5 Conclusion

In summary, our findings point out that exercise-induced inflammatory responses could alter emotion perception, manifesting primarily as diminished recognition rates of emotional content of social interactions. This effect can be attributed to a diminished sensitivity to emotion-specific cues across all emotional categories. While changes in visual scanning behavior were observed, they cannot account for the altered perceptual performance, as those effects were not observed irrespective of the emotion category. Thus, the effects of inflammation on perception likely stem from changes in neural activity within brain regions involved in emotion processing and mood regulation, such as limbic and cortical areas, which are susceptible to fluctuations in blood cytokine concentrations (Dantzer et al., 2008; Lasselin et al., 2018).

To the best of our knowledge, this study provides first evidence that exercise-induced low-grade inflammation potentially might impair the accurate and reliable comprehension of emotional information conveyed through perceived body language during social interactions. However, further research is warranted to gain a more comprehensive understanding of inflammation-induced changes in social cognition, including their implications for both healthy populations and individuals with neuropsychiatric disorders such as depression, but also lifestyle-related diseases, like diabetes-type 2 and arteriosclerosis.

## Funding statement

The research was funded by the Deutsche Forschungsgemeinschaft (DFG; German Research Foundation) International Research Training Group (IRTG, 1901), “The Brain in Action”, under a grant for J.K.

## Ethics approval statement

Ethical approval was provided by the Ethics Commission of the FB06, JLU Giessen.

## Patient consent statement

Following the guidelines of the Declaration of Helsinki, written informed consent was obtained from all participants before participation in the study**.**

## Statement

During the preparation of this work the author(s) did not use generative AI and AI-assisted technologies.

## CRediT authorship contribution statement

**Johannes Keck:** Writing – review & editing, Writing – original draft, Visualization, Validation, Software, Resources, Methodology, Investigation, Funding acquisition, Formal analysis, Data curation, Conceptualization. **Celine Honekamp:** Writing – review & editing, Writing – original draft, Visualization, Validation, Software, Resources, Methodology, Investigation, Formal analysis, Data curation, Conceptualization. **Kristina Gebhardt:** Writing – review & editing, Writing – original draft, Validation, Resources, Investigation, Formal analysis, Data curation, Conceptualization. **Svenja Nolte:** Writing – review & editing, Writing – original draft, Validation, Resources, Investigation, Data curation, Conceptualization. **Marcel Linka:** Writing – review & editing, Writing – original draft, Software, Resources, Investigation, Formal analysis, Data curation, Conceptualization. **Benjamin de Haas:** Writing – review & editing, Writing – original draft, Validation, Supervision, Project administration, Conceptualization. **Jörn Munzert:** Writing – review & editing, Writing – original draft, Validation, Supervision, Project administration, Funding acquisition, Conceptualization. **Karsten Krüger:** Writing – review & editing, Writing – original draft, Validation, Supervision, Resources, Project administration, Funding acquisition, Conceptualization. **Britta Krüger:** Writing – review & editing, Writing – original draft, Visualization, Validation, Supervision, Resources, Project administration, Methodology, Investigation, Funding acquisition, Formal analysis, Data curation, Conceptualization.

## Declaration of competing interest

The authors declare that they have no known competing financial interests or personal relationships that could have appeared to influence the work reported in this paper.

## Data Availability

Data will be made available on request.

## References

[bib2] Alaerts K., Nackaerts E., Meyns P., Swinnen S.P., Wenderoth N. (2011). Action and emotion recognition from point light displays: An investigation of gender differences. PLoS One.

[bib3] Atkinson A.P., Dittrich W.H., Gemmell A.J., Young A.W. (2004). Emotion perception from dynamic and static body expressions in point-light and full-light displays. Perception.

[bib5] Bachmann J., Munzert J., Krüger B. (2018). Neural underpinnings of the perception of emotional states derived from biological human motion: a review of neuroimaging research. Front. Psychol..

[bib6] Bachmann J., Zabicki A., Munzert J., Krüger B. (2020). Emotional expressivity of the observer mediates recognition of affective states from human body movements. Cognit. Emot..

[bib7] Balter L.J.T., Hulsken S., Aldred S., Drayson M.T., Higgs S., Veldhuijzen van Zanten J.J.C.S., Raymond J.E., Bosch J.A. (2018). Low-grade inflammation decreases emotion recognition – evidence from the vaccination model of inflammation. Brain Behav. Immun..

[bib8] Benson S., Brinkhoff A., Lueg L., Roderigo T., Kribben A., Wilde B., Witzke O., Engler H., Schedlowski M., Elsenbruch S. (2017). Effects of acute systemic inflammation on the interplay between sad mood and affective cognition. Transl. Psychiatry.

[bib9] Bollen J., Trick L., Llewellyn D., Dickens C. (2017). The effects of acute inflammation on cognitive functioning and emotional processing in humans: a systematic review of experimental studies. J. Psychosom. Res..

[bib11] Bruunsgaard H., Galbo H., Halkjaer-Kristensen J., Johansen T.L., MacLean D.A., Pedersen B.K. (1997). Exercise-induced increase in serum interleukin-6 in humans is related to muscle damage. J. Physiol..

[bib15] Dantzer R., Kelley K.W. (2007). Twenty years of research on cytokine-induced sickness behavior. Brain Behav. Immun..

[bib16] Dantzer R., Konsman J.-P., Bluthé R.-M., Kelley K.W. (2000). Neural and humoral pathways of communication from the immune system to the brain: parallel or convergent?. Auton. Neurosci..

[bib17] Dantzer R., O'Connor J.C., Freund G.G., Johnson R.W., Kelley K.W. (2008). From inflammation to sickness and depression: when the immune system subjugates the brain. Nat. Rev. Neurosci..

[bib18] de Gelder B. (2006). Towards the neurobiology of emotional body language. Nat. Rev. Neurosci..

[bib19] de Gelder B., De Borst A.W., Watson R. (2015). The perception of emotion in body expressions: emotional body perception. Wiley Interdisciplinary Rev.: Cognit. Sci..

[bib20] de Haas B., Iakovidis A.L., Schwarzkopf D.S., Gegenfurtner K.R. (2019). Individual differences in visual salience vary along semantic dimensions. Proc. Nat. Acad. Sci. USA.

[bib74] Dowlati Y, Herrmann N, Swardfager W, Liu H, Sham L, Reim EK, Lanctôt KL (2010 Mar 1). A meta-analysis of cytokines in major depression. Biol. Psychiatr..

[bib22] Eisenberger N.I., Inagaki T.K., Mashal N.M., Irwin M.R. (2010). Inflammation and social experience: an inflammatory challenge induces feelings of social disconnection in addition to depressed mood. Brain Behav. Immun..

[bib23] Eisenberger N.I., Inagaki T.K., Rameson L.T., Mashal N.M., Irwin M.R. (2009). An fMRI study of cytokine-induced depressed mood and social pain: the role of sex differences. Neuroimage.

[bib24] Elenkov I.J. (2008). Neurohormonal-cytokine interactions: implications for inflammation, common human diseases and well-being. Neurochem. Int..

[bib25] Elliott R., Zahn R., Deakin J.F., Anderson I.M. (2011). Affective cognition and its disruption in mood disorders. Neuropsychopharmacology.

[bib26] Engler H., Benson S., Wegner A., Spreitzer I., Schedlowski M., Elsenbruch S. (2016). Men and women differ in inflammatory and neuroendocrine responses to endotoxin but not in the severity of sickness symptoms. Brain Behav. Immun..

[bib28] Gallese V. (2007). Embodied simulation: from mirror neuron systems to interpersonal relations. Novartis Found. Symp..

[bib29] Gebhardt K., Hebecker A., Honekamp C., Nolte S., Bartkuhn M., Wilhelm J., Klatt S., Weyh C., Sommer N., Krüger K. (2024). Respiratory and metabolic responses of CD4+ T cells to acute exercise and their association with cardiorespiratory fitness. Med. Sci. Sports Exerc..

[bib77] Girden E.R. (1992). ANOVA: Repeated Measures.

[bib30] Grigoleit J.-S., Kullmann J.S., Wolf O.T., Hammes F., Wegner A., Jablonowski S., Engler H., Gizewski E., Oberbeck R., Schedlowski M. (2011). Dose-dependent effects of endotoxin on neurobehavioral functions in humans. PLoS One.

[bib31] Hansson L.S., Axelsson J., Petrovic P., Paues Göranson S., Olsson M.J., Lekander M., Lasselin J. (2021). Regulation of emotions during experimental endotoxemia: a pilot study. Brain Behav. Immun..

[bib32] Harrison N.A., Voon V., Cercignani M., Cooper E.A., Pessiglione M., Critchley H.D. (2016). A neurocomputational account of how inflammation enhances sensitivity to punishments versus rewards. Biol. Psychiatr..

[bib33] Hodgman C.F., Hunt R.M., Crane J.C., Elzayat M.T., LaVoy E.C. (2023). A scoping review on the effects of physical exercise and fitness on peripheral leukocyte energy metabolism in humans. Exerc. Immunol. Rev..

[bib76] Kadoglou NP, Iliadis F, Angelopoulou N, Perrea D, Ampatzidis G, Liapis CD, Alevizos M (2007 Dec). The anti-inflammatory effects of exercise training in patients with type 2 diabetes mellitus. Eur. J. Cardiovasc. Prev. Rehabil..

[bib34] Keck J., Zabicki A., Bachmann J., Munzert J., Krüger B. (2022). Decoding spatiotemporal features of emotional body language in social interactions. Sci. Rep..

[bib36] Konsman J.P., Parnet P., Dantzer R. (2002). Cytokine-induced sickness behaviour: mechanisms and implications. Trends Neurosci..

[bib37] Kotulla S., Elsenbruch S., Roderigo T., Brinkhoff A., Wegner A., Engler H., Schedlowski M., Benson S. (2018). Does human experimental endotoxemia impact negative cognitions related to the self?. Front. Behav. Neurosci..

[bib40] Kullmann J.S., Grigoleit J.-S., Wolf O.T., Engler H., Oberbeck R., Elsenbruch S., Forsting M., Schedlowski M., Gizewski E.R. (2014). Experimental human endotoxemia enhances brain activity during social cognition. Soc. Cognit. Affect Neurosci..

[bib41] Lasselin J., Lekander M., Axelsson J., Karshikoff B. (2018). Sex differences in how inflammation affects behavior: what we can learn from experimental inflammatory models in humans. Front. Neuroendocrinol..

[bib75] Maes M, Meltzer HY, Bosmans E, Bergmans R, Vandoolaeghe E, Ranjan R, Desnyder R (1995 Aug 18). Increased plasma concentrations of interleukin-6, soluble interleukin-6, soluble interleukin-2 and transferrin receptor in major depression. J. Affect. Disord..

[bib42] Maier S.F., Watkins L.R. (1998). Cytokines for Psychologists: Implications of Bidirectional Immune-to- Brain Communication for Understanding Behavior, Mood, and Cognition.

[bib44] Miller A.H., Maletic V., Raison C.L. (2009). Inflammation and its discontents: the role of cytokines in the pathophysiology of major depression. Biol. Psychiatr..

[bib45] Moieni M., Irwin M.R., Jevtic I., Breen E.C., Eisenberger N.I. (2015). Inflammation impairs social cognitive processing: a randomized controlled trial of endotoxin. Brain Behav. Immun..

[bib46] Moieni M., Irwin M.R., Jevtic I., Olmstead R., Breen E.C., Eisenberger N.I. (2015). Sex differences in depressive and socioemotional responses to an inflammatory challenge: implications for sex differences in depression. Neuropsychopharmacology.

[bib47] Muscatell K.A., Dedovic K., Slavich G.M., Jarcho M.R., Breen E.C., Bower J.E., Irwin M.R., Eisenberger N.I. (2016). Neural mechanisms linking social status and inflammatory responses to social stress. Soc. Cognit. Affect Neurosci..

[bib48] Nakao A. (2014). Temporal regulation of cytokines by the circadian clock. J. Immunol. Res..

[bib49] Paulus C.M. (2009). Der Saarbrucker Personlichkeitsfragebogen SPF (IRI) zur Messung von Empathie: Psychometrische Evaluation der deutschen Version des Interpersonal Reactivity Index. http://psydok.psycharchives.de/jspui/handle/20.500.11780/3343.

[bib50] Paulsen G., Mikkelsen U.R., Raastad T., Peake J.M. (2012). Leucocytes, cytokines and satellite cells: what role do they play in muscle damage and regeneration following eccentric exercise?. Exerc. Immunol. Rev..

[bib51] Peake J.M., Della Gatta P., Suzuki K., Nieman D.C. (2015). Cytokine expression and secretion by skeletal muscle cells: regulatory mechanisms and exercise effects. Exerc. Immunol. Rev..

[bib52] Peake J.M., Neubauer O., Della Gatta P.A., Nosaka K. (2017). Muscle damage and inflammation during recovery from exercise. J. Appl. Physiol..

[bib53] Peake J.M., Suzuki K., Wilson G., Hordern M., Nosaka K., Mackinnon L., Coombes J.S. (2005). Exercise-induced muscle damage, plasma cytokines, and markers of neutrophil activation. Med. Sci. Sports Exerc..

[bib55] Pedersen B.K., Febbraio M.A. (2008). Muscle as an endocrine organ: focus on muscle-derived interleukin-6. Physiol. Rev..

[bib57] Poyo Solanas M., Vaessen M.J., De Gelder B. (2020). The role of computational and subjective features in emotional body expressions. Sci. Rep..

[bib58] Proschinger S., Freese J. (2019). Neuroimmunological and neuroenergetic aspects in exercise-induced fatigue. Exerc. Immunol. Rev..

[bib59] Rindermann H. (2009).

[bib60] Schedlowski M., Engler H., Grigoleit J.-S. (2014). Endotoxin-induced experimental systemic inflammation in humans: a model to disentangle immune-to-brain communication. Brain Behav. Immun..

[bib61] Shek P.N., Shephard R.J. (1998). Physical exercise as a human model of limited inflammatory response. Can. J. Physiol. Pharmacol..

[bib62] Smith L.L. (2000). Cytokine hypothesis of overtraining: a physiological adaptation to excessive stress?. Med. Sci. Sports Exerc..

[bib63] Steensberg A., Keller C., Starkie R.L., Osada T., Febbraio M.A., Pedersen B.K. (2002). IL-6 and TNF-α expression in, and release from, contracting human skeletal muscle. Am. J. Physiol. Endocrinol. Metabol..

[bib64] Suslow T., Hußlack A., Kersting A., Bodenschatz C.M. (2020). Attentional biases to emotional information in clinical depression: a systematic and meta-analytic review of eye tracking findings. J. Affect. Disord..

[bib65] Suzuki K. (2018). Cytokine response to exercise and its modulation. Antioxidants.

[bib78] Todd RM, Cunningham WA, Anderson AK, Thompson E (2012 Jul). Affect-biased attention as emotion regulation. Trends Cognit. Sci..

[bib68] Turnbull A.V., Rivier C.L. (1999). Regulation of the hypothalamic-pituitary-adrenal Axis by cytokines: actions and mechanisms of action. Physiol. Rev..

[bib69] Van Den Stock J., Righart R., De Gelder B. (2007). Body expressions influence recognition of emotions in the face and voice. Emotion.

[bib72] Wegner A., Benson S., Rebernik L., Spreitzer I., Jäger M., Schedlowski M., Elsenbruch S., Engler H. (2017). Sex differences in the pro-inflammatory cytokine response to endotoxin unfold *in vivo* but not *ex vivo* in healthy humans. Innate Immun..

[bib73] Wright C.E., Strike P.C., Brydon L., Steptoe A. (2005). Acute inflammation and negative mood: mediation by cytokine activation. Brain Behav. Immun..

