# Supplementary material for: Exercise-induced inflammation alters the perception and visual exploration of emotional interactions
Source: Brain Behav Immun Health. 2024 Jun 11;39:100806. doi: 10.1016/j.bbih.2024.100806 (PMC11225855; doi:10.1016/j.bbih.2024.100806)
Supplement: Multimedia component 1 [file mmc1.docx]

**Supplementary Information**

**Descriptives**

*Markers of muscle damage and inflammation*

| Condition | Time | Mean | SD | N |
| --- | --- | --- | --- | --- |
| **IL6** | | | | |
| Inf | T0 | 2.067 | 2.142 | 17 |
|  | T1 | 2.749 | 2.580 | 17 |
|  | T3 | 3.212 | 2.340 | 17 |
|  | T24 | 2.638 | 2.560 | 17 |
| Con | T0 | 2.280 | 2.442 | 17 |
|  | T1 | 2.918 | 2.581 | 17 |
|  | T3 | 2.326 | 2.164 | 17 |
|  | T24 | 2.137 | 2.222 | 17 |
| **CRP** | | | | |
| Inf | T0 | 412294.804 | 244192.107 | 17 |
|  | T1 | 496966.376 | 327038.456 | 17 |
|  | T3 | 613349.037 | 378204.244 | 17 |
|  | T24 | 1.005e +6 | 749302.146 | 17 |
| Con | T0 | 441896.644 | 305014.274 | 17 |
|  | T1 | 452727.704 | 379445.666 | 17 |
|  | T3 | 477362.861 | 337934.001 | 17 |
|  | T24 | 549022.169 | 514314.213 | 17 |
| **Myoglobin** | | | | |
| Inf | T0 | 3577.198 | 3762.788 | 19 |
|  | T1 | 8632.295 | 9278.289 | 19 |
|  | T3 | 12047.580 | 12911.428 | 19 |
|  | T24 | 5349.283 | 5479.160 | 19 |
| Con | T0 | 3569.581 | 3974.854 | 19 |
|  | T1 | 7126.235 | 8425.516 | 19 |
|  | T3 | 6948.435 | 8765.144 | 19 |
|  | T24 | 3704.356 | 3871.170 | 19 |
| **TNF Alpha** | | | | |
| Inf | T0 | 2.372 | 1.417 | 19 |
|  | T1 | 2.662 | 1.555 | 19 |
|  | T3 | 2.663 | 1.400 | 19 |
|  | T24 | 2.471 | 1.493 | 19 |
| Con | T0 | 2.288 | 1.328 | 19 |
|  | T1 | 2.590 | 1.359 | 19 |
|  | T3 | 2.347 | 1.238 | 19 |
|  | T24 | 2.204 | 1.420 | 19 |
| **MCP1** | | | | |
| Inf | T0 | 317.879 | 106.069 | 17 |
|  | T1 | 457.445 | 185.292 | 17 |
|  | T3 | 409.207 | 139.430 | 17 |
|  | T24 | 277.143 | 60.459 | 17 |
| Con | T0 | 297.251 | 120.503 | 17 |
|  | T1 | 397.565 | 139.246 | 17 |
|  | T3 | 331.731 | 114.416 | 17 |
|  | T24 | 288.766 | 87.389 | 17 |

*Influence of exercise-induced inflammation on emotion recognition*

| Emotion | Condition | Mean | SD | N |
| --- | --- | --- | --- | --- |
| **Emotion Recognition** | | | | |
| Happiness | Inf | 0.895 | 0.114 | 19 |
|  | Con | 0.904 | 0.089 | 19 |
|  | Rest | 0.921 | 0.098 | 19 |
| Affection | Inf | 0.768 | 0.138 | 19 |
|  | Con | 0.798 | 0.116 | 19 |
|  | Rest | 0.807 | 0.155 | 19 |
| Sadness | Inf | 0.851 | 0.113 | 19 |
|  | Con | 0.842 | 0.127 | 19 |
|  | Rest | 0.890 | 0.108 | 19 |
| Anger | Inf | 0.864 | 0.115 | 19 |
|  | Con | 0.877 | 0.085 | 19 |
|  | Rest | 0.921 | 0.076 | 19 |
| **Intensity** | | | | |
| Happiness | Inf | 2.096 | 0.243 | 19 |
|  | Con | 1.991 | 0.269 | 19 |
|  | Rest | 1.943 | 0.253 | 19 |
| Affection | Inf | 1.671 | 0.326 | 19 |
|  | Con | 1.575 | 0.319 | 19 |
|  | Rest | 1.535 | 0.364 | 19 |
| Sadness | Inf | 1.430 | 0.325 | 19 |
|  | Con | 1.425 | 0.298 | 19 |
|  | Rest | 1.399 | 0.326 | 19 |
| Anger | Inf | 1.553 | 0.253 | 19 |
|  | Con | 1.579 | 0.336 | 19 |
|  | Rest | 1.443 | 0.363 | 19 |
| **Confidence** | | | | |
| Happiness | Inf | 3.425 | 0.767 | 19 |
|  | Con | 3.268 | 0.840 | 19 |
|  | Rest | 3.566 | 0.742 | 19 |
| Affection | Inf | 2.855 | 0.731 | 19 |
|  | Con | 3.004 | 0.821 | 19 |
|  | Rest | 2.732 | 0.956 | 19 |
| Sadness | Inf | 2.601 | 1.080 | 19 |
|  | Con | 2.693 | 0.921 | 19 |
|  | Rest | 2.338 | 1.115 | 19 |
| Anger | Inf | 2.614 | 1.117 | 19 |
|  | Con | 2.868 | 1.037 | 19 |
|  | Rest | 2.724 | 1.012 | 19 |
| **Sensitivity** | | | | |
| Happiness | Inf | 2.671 | 0.607 | 19 |
|  | Con | 2.895 | 0.519 | 19 |
|  | Rest | 3.017 | 0.539 | 19 |
| Affection | Inf | 2.565 | 0.519 | 19 |
|  | Con | 2.648 | 0.562 | 19 |
|  | Rest | 2.932 | 0.513 | 19 |
| Sadness | Inf | 2.810 | 0.473 | 19 |
|  | Con | 2.823 | 0.581 | 19 |
|  | Rest | 3.185 | 0.447 | 19 |
| Anger | Inf | 2.799 | 0.556 | 19 |
|  | Con | 2.936 | 0.470 | 19 |
|  | Rest | 3.164 | 0.460 | 19 |

*Influence of exercise-induced inflammation on eye movements*

| Emotion | Condition | Mean | SD | N |
| --- | --- | --- | --- | --- |
| **First Fixation Latency** | | | | |
| Happiness | Inf | 329.891 | 61.351 | 19 |
|  | Con | 297.277 | 42.680 | 19 |
|  | Rest | 307.848 | 48.049 | 19 |
| Affection | Inf | 322.299 | 107.203 | 19 |
|  | Con | 326.868 | 77.748 | 19 |
|  | Rest | 288.236 | 43.616 | 19 |
| Sadness | Inf | 313.302 | 103.120 | 19 |
|  | Con | 295.091 | 55.092 | 19 |
|  | Rest | 338.305 | 65.681 | 19 |
| Anger | Inf | 314.992 | 43.784 | 19 |
|  | Con | 303.326 | 60.275 | 19 |
|  | Rest | 314.157 | 60.598 | 19 |
| **First Fixation Duration** | | | | |
| Happiness | Inf | 572.757 | 0.243 | 19 |
|  | Con | 620.119 | 0.269 | 19 |
|  | Rest | 401.829 | 0.253 | 19 |
| Affection | Inf | 638.219 | 0.326 | 19 |
|  | Con | 577.202 | 0.319 | 19 |
|  | Rest | 626.141 | 0.364 | 19 |
| Sadness | Inf | 389.548 | 0.325 | 19 |
|  | Con | 560.791 | 0.298 | 19 |
|  | Rest | 505.054 | 0.326 | 19 |
| Anger | Inf | 596.961 | 0.253 | 19 |
|  | Con | 359.605 | 0.336 | 19 |
|  | Rest | 580.938 | 0.363 | 19 |
| **Dwell Time** | | | | |
| Happiness | Inf | 3380.691 | 152.453 | 19 |
|  | Con | 3415.807 | 161.111 | 19 |
|  | Rest | 3355.009 | 154.006 | 19 |
| Affection | Inf | 3356.693 | 199.195 | 19 |
|  | Con | 3400.787 | 122.982 | 19 |
|  | Rest | 3500.443 | 101.388 | 19 |
| Sadness | Inf | 3382.342 | 121.521 | 19 |
|  | Con | 3435.389 | 102.597 | 19 |
|  | Rest | 3387.718 | 193.050 | 19 |
| Anger | Inf | 3453.320 | 157.766 | 19 |
|  | Con | 3389.044 | 157.961 | 19 |
|  | Rest | 3413.881 | 146.226 | 19 |
| **Average Glance Duration** | | | | |
| Happiness | Inf | 821.155 | 415.700 | 19 |
|  | Con | 800.418 | 336.446 | 19 |
|  | Rest | 628.954 | 263.525 | 19 |
| Affection | Inf | 793.366 | 347.908 | 19 |
|  | Con | 845.642 | 378.451 | 19 |
|  | Rest | 828.794 | 353.297 | 19 |
| Sadness | Inf | 605.228 | 187.663 | 19 |
|  | Con | 739.316 | 277.817 | 19 |
|  | Rest | 798.805 | 379.125 | 19 |
| Anger | Inf | 798.201 | 281.089 | 19 |
|  | Con | 574.002 | 171.974 | 19 |
|  | Rest | 730.099 | 327.388 | 19 |
| **Number of Fixations** | | | | |
| Happiness | Inf | 5.427 | 1.608 | 19 |
|  | Con | 5.702 | 1.500 | 19 |
|  | Rest | 6.671 | 1.572 | 19 |
| Affection | Inf | 5.798 | 1.482 | 19 |
|  | Con | 5.512 | 1.362 | 19 |
|  | Rest | 5.447 | 1.439 | 19 |
| Sadness | Inf | 6.570 | 1.477 | 19 |
|  | Con | 6.008 | 1.353 | 19 |
|  | Rest | 5.596 | 1.511 | 19 |
| Anger | Inf | 5.395 | 1.308 | 19 |
|  | Con | 6.763 | 1.533 | 19 |
|  | Rest | 5.965 | 1.506 | 19 |
